# ICARE Radon Calibration Device

**DOI:** 10.6028/jres.095.017

**Published:** 1990

**Authors:** P. Zettwoog

**Affiliations:** Centre d’Etudes Nucléaires de Fontenay-aux-Roses, CEN/FAR, BP 6, 92265, Fontenay-aux-Roses Cedex, France

**Keywords:** calibration, decay products, metrology, ^222^Rn, standard

## Abstract

An aerodynamic calibration device called ICARE is briefly described. ICARE is currently being used at the Nuclear Research Center of Saclay in France for the certification of instruments employed in measurement of artificial radioactive particulate airborne contamination. ICARE is essentially a blower. Aerosols calibrated in size and labelled with ^137^Cs or ^239^Pu are injected in the mainstream of the blower upstream of the test section.

To extend ICARE’s field of application to the case of instruments employed in measurement of ^222^Rn and of its decay products, a new line of injection has been designed and is under construction, in the frame of a contract financed by the European Community Commission. The main effort is presently oriented to-ward the development of a reliable ^222^Rn source, based on a solid deposit of ^226^Ra on organic fibers, and of a reference method for the measurement of ^222^Rn activity per unit volume of air. This reference is based on a modified version of standard containers being used in the French reference method for calibrated measurements of ^133^Xe and ^85^Kr by gamma spectroscopy.

## 1. Introduction

In France, the Technical Centre for Ratification of Radiation Protection Instruments is responsible for approval of all instruments used by radiation protection officers for surveillance of ambient radiation or for dosimetry of ionizing radiation.

In the case of equipment designed for measurement of gaseous or particulate contamination of the atmosphere, special test methods are needed for aerodynamic certification and calibration.

An aerodynamic calibration device using standard radioactive aerosols called ICARE (Installation de Calibration à 1’Aide d’Aérosols Radioactifs Etalons) has been designed for this purpose by J. Charuau and M. Ammerich [1]. It is currently being used for certification of instruments employed in measurement of artificial, radioactive, particulate contamination (alpha and beta emitters).

This paper describes the new procedures that have recently been used to adapt ICARE to ^222^Rn and decay products measurements.

## 2. Description of ICARE

**Figure 1:**
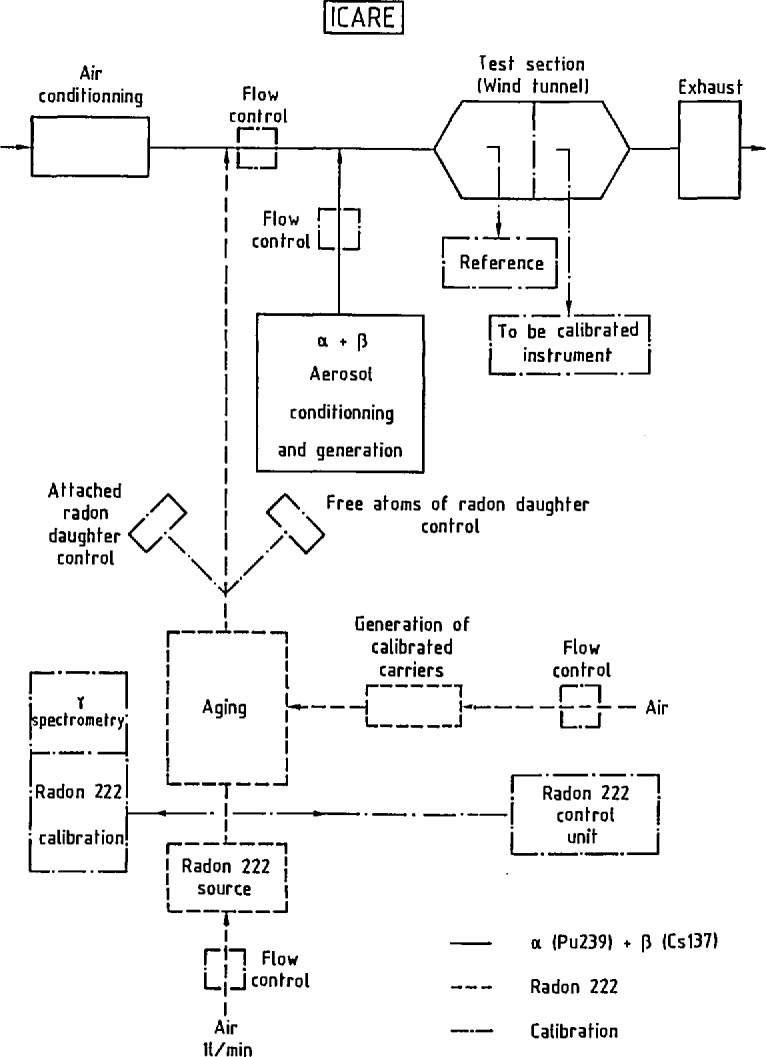
Description of ICARE.

**Figure 2:**
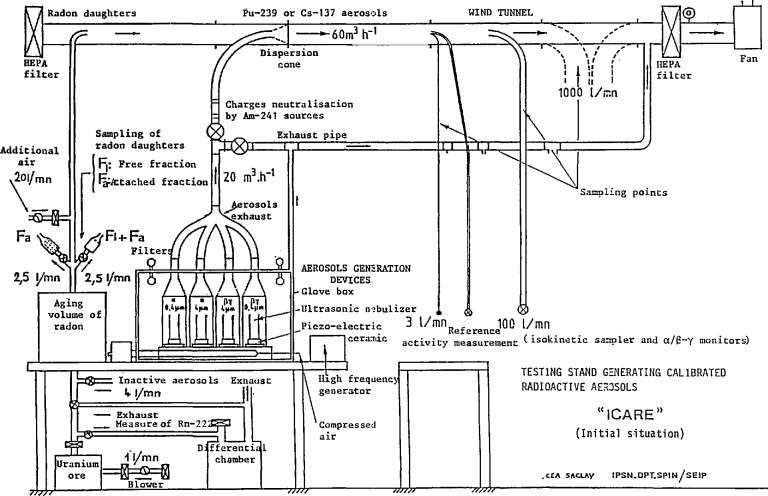
Schematic diagram of ICARE.

**Figure 3:**
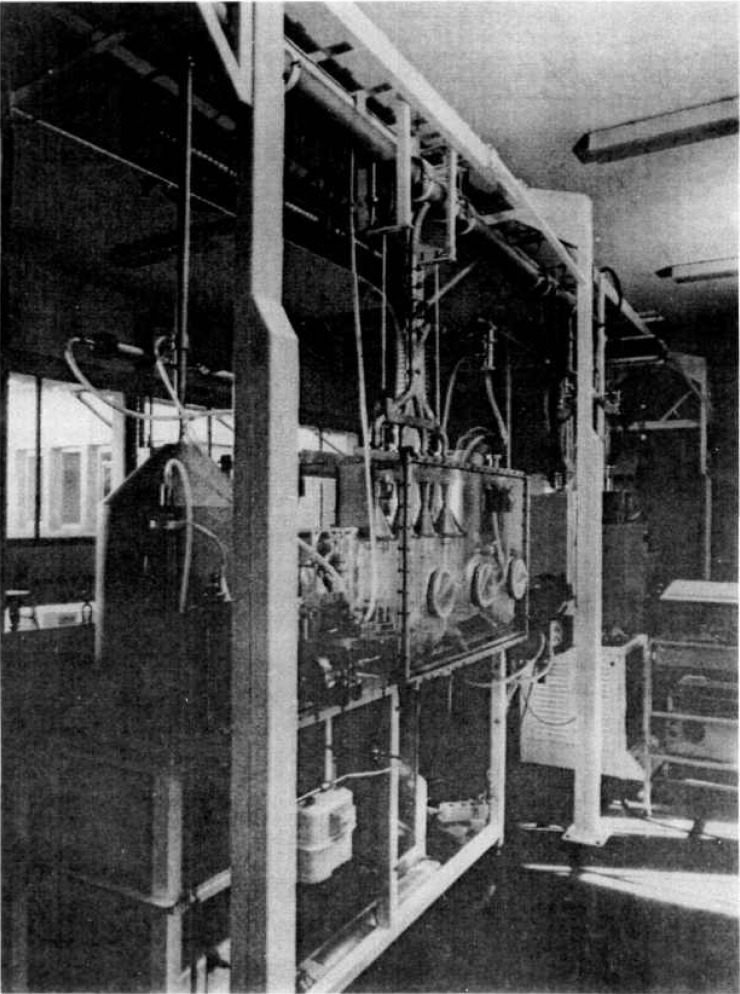
General view of device.

**Figure 4:**
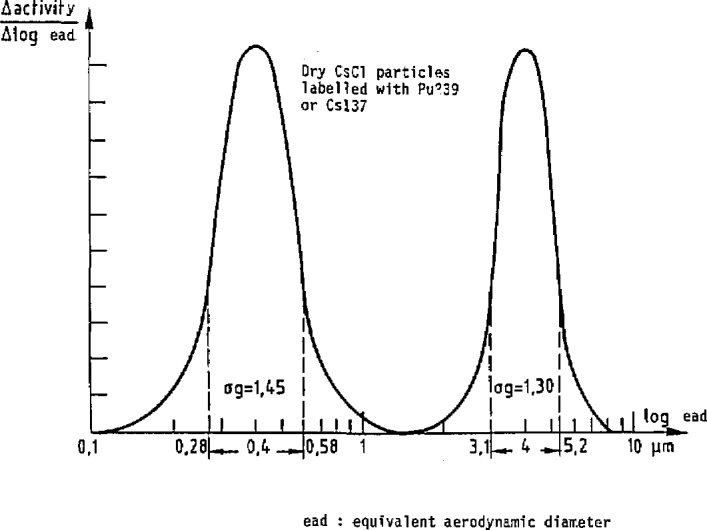
Particle size distribution of test aerosols.

The essential characteristic of this device is that it operates under dynamic conditions. The device is a blower (figs. 1, 2, and 3) linked to the peripheral air-conditioning units. ICARE offers test conditions that are perfectly designed, both in terms of aerodynamics and of aerosol physics for the characterization of radioactive aerosols monitoring systems. Calibrated aerosols of CsCl with size distribution centered on 0.4 and 4 *μ*m (fig. 4), and radioactively labelled with ^137^Cs and ^239^Pu are generated and injected in the mainstream of the blower. The dilution factor of the injection line flow by the mainstream is kept constant. The blower concept ensures that exactly the same atmosphere is delivered by the sampling line used for the apparatus and by the sampling line used for the apparatus to be certificated.

In the blower concept, the final precision of the calibration provided depends mainly on the precision of the various instruments used to measure air mass flow (fig. 1) linked to the sampling and injection lines and to the mainstream, and also on the measurement of artificial radioactivity by alpha and beta reference spectrometry.

ICARE was initially equipped with an injection line for ^222^Rn and its decay products, which may or may not attach to natural atmospheric condensation nuclei or to test aerosols. The aim was only to verify that, with low-level airborne ^239^Pu contamination, the measurement instruments were able to discriminate between alpha emissions from ^239^Pu and those of short-lived polonium isotopes naturally present in the laboratories. As the aim was not to calibrate instruments for the measurement of ^222^Rn and its decay products, there was no need for stable radon generation. In fact, the stability of ^222^Rn emission from the source (uranium ore) was large and inadequate for calibrating instruments for the measurement of ^22^Rn. In addition there was no control over the concentration and particle size of the aerosols bearing radon decay products, and hence no possibility of free choice of the equilibrium factor and of the unattached fraction of the decay products.

This is why, in a separate contract financed by the European Community Commission, a new Une for injection of radon and its decay products has been designed and is being developed (ICARE Radon). The aim of the first phase (1988–1989) is to produce an atmosphere with a perfectly defined and stable radon 222 activity per unit volume. The second phase (1989–1990) will be devoted to development of an atmosphere in which the activities per unit volume of short-lived radon decay products and the particle size distribution will also be perfectly defined and stable. A description will only be given of the first phase, which is the subject of the thesis by M. Guelin [2].

## 3. The Radon Source

**Figure 5:**
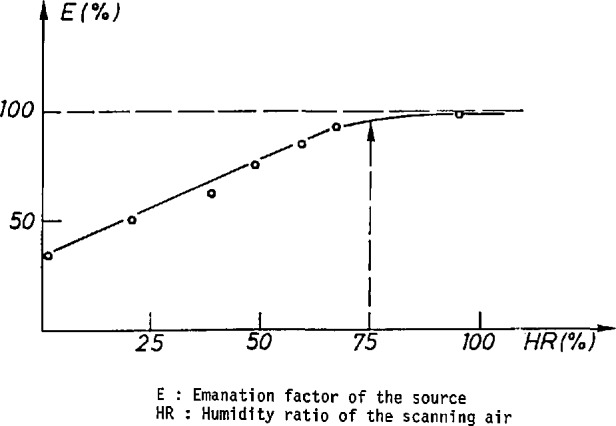
Emanation factor of the source as a function of the scanning air humidity ratio.

To obtain a stable, reliable radon source we prefer to package the radium in the form of a solid deposit fixed to an appropriate support, rather than solubilize ions in a liquid. Sources in which ^226^Ra is adsorbed by ion-exchange resins have been described and are commercially available. Following a laboratory study of the binding of soluble radium to organic fibers impregnated with manganese oxides, we found that these fibers constitute the ideal support for a solid ^222^Rn source. Indeed, we found that the emanation factor of radon emitted by such a source was about 100%, and was stable for scanning air humidity ratios greater than 80% fibers (fig. 5) [4]. The pressure loss in the fibers is negligible.

**Figure 6:**
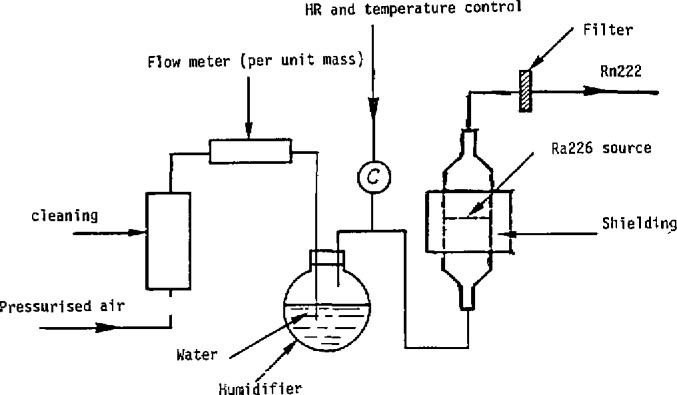
Diagram showing radon source.

A diagram of the ^222^Rn source is shown in figure 6. This device comprises an air-supply circuit with an on-line humidifier, flow rate controls on all air inlets, and an ionization chamber used to control the stability of generation and scanning. The radon injection line ends at the main pipeline of the blower upline from the test unit and from the unit for injection of artificial aerosols. Stirring devices ensure good mixing of the air from the radon injection line and the air from the blower.

The activity of the solid source of ^226^Ra is measured by gamma spectrometry, after establishment of equilibrium between the ^226^Ra and ^214^Bi. Emission of ^222^Rn by the source expressed in atom · s^−1^ is equal to the activity of the ^226^Ra of the source expressed in Bq. The activity per unit volume of radon in the air delivered is equal to the product of the decay constant of ^222^Rn and the source intensity expressed in atom · s^−1^, divided by the scanning air flow rate, which is measured with a reference flow meter. Hence:

$$A_{\text{Rn}}^{v}=\frac{\lambda 222\;A_{\text{ra}}}{Q_{v}}$$ (1)

where:

$A_{\text{Rn}}^{v}$ = activity of ^222^Rn per unit volume (Bq · m^−3^) in the scanning air
λ222 = 2.1×10^−6^s^−1^
*A*_ra_ = activity of the radium source (Bq)
*Q*_v_ =flow rate of scanning air (m^3^·s^−1^).

## 4. Development of a Reference Method for ^222^Rn Activity per Unit Volume Measurement

**Figure 7:**
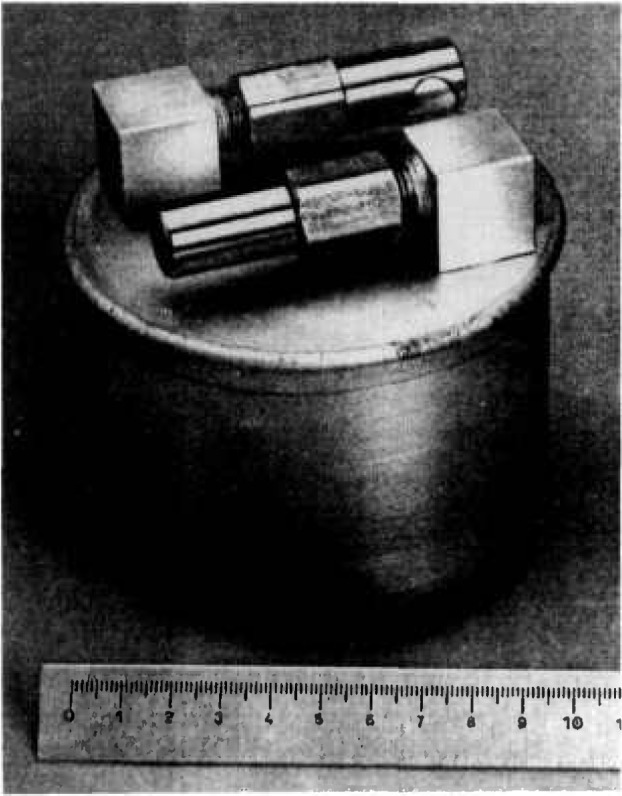
Standard Xe Kr containers.

It is possible in theory to apply eq (1) in order to define $A_{\text{Rn}}^{v}$ in the injection line. It has seemed to us unwise to rely uniquely on eq (1) for definition of ^222^Rn activity per unit volume in the injection line, since this would involve the assumption that the radon emanation factor is strictly equal to 100%. This is why we have sought to develop a reference method for measurement of the airborne activity per unit volume, which may also by used in other situations. We took as our starting point the fact that there already exist standard containers for the measurement by gamma spectroscopy of the activity of ^133^Xe and ^85^Kr per unit volume of atmosphere (fig. 7).

**Figure 8:**
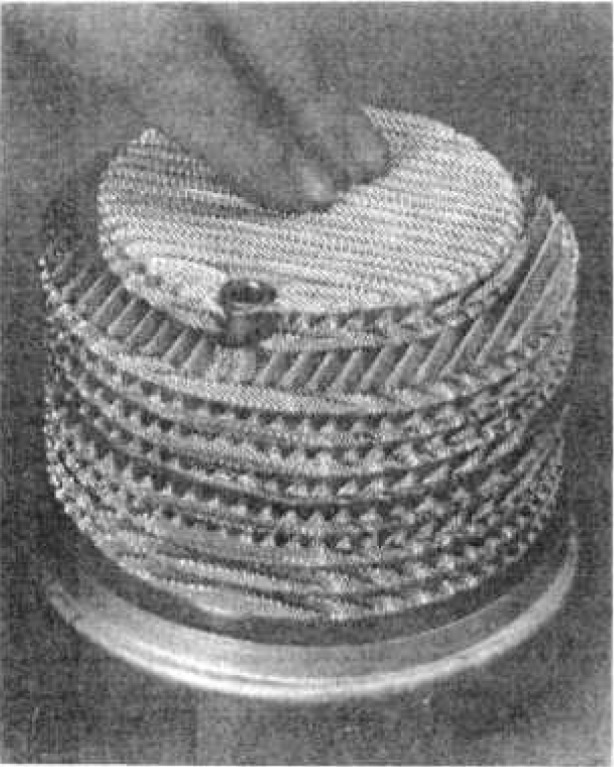
Internal structure of the ^222^Rn container.

**Figure 9:**
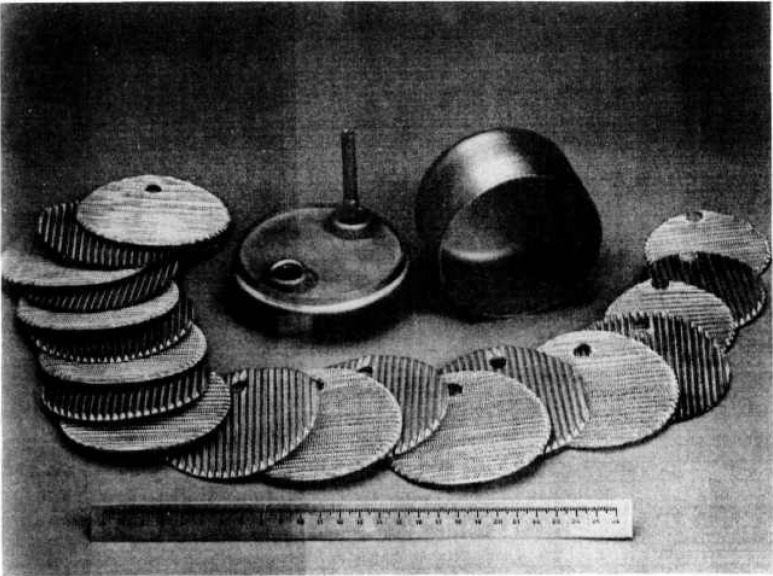
Display of the metal mesh structure.

Direct use of this type of container involves the difficulty that gamma spectrometry measurement of ^222^Rn can only be achieved by examining gamma rays from one of the short-lived decay products, ^214^Bi, considered to be in equilibrium with ^222^Rn after 3 h. The difficulty is that since a non-gaseous decay product is involved at room temperature, the atoms formed by decay bind to the walls of the container and hence distribution of activity in the container volume is no longer homogeneous; thus, it is not easy to assess. To remedy this situation, it was decided to fill the inside of the container with a metal mesh structure of low gamma radiation absorption. This structure comprises light alloy meshes which allow for deposition of ^214^Bi within the container space to be uniform at least on a scale of the mesh size of the metal structure, i.e., a few millimeters (figs. 8 and 9). This new device has been patented [3].

The container intended for regular measurement of the airborne activity per unit volume in the radon injection line, the stability of which is guaranteed by continuous monitoring of the injection air flow rate and by the response of the ionization chamber serving as source monitor, will have a lower limit sensitivity of about 2,000 Bq·m^−3^ under laboratory conditions at the Nuclear Research Center of Saclay. With the gamma spectrometer used, the sampled air volume is about 500 cm^3^

The activity per unit volume in the test unit is obtained from that of the injection line by dividing the factor defining the dilution produced by the mainstream of the blower. The accuracy of this dilution factor is that obtained with reference flow meters. The homogeneity of the mixture is checked beforehand by injecting helium as a tracer in the radon injection line and examining the spatial distribution of the helium concentration in the test section with a mass spectrometer. In the reference sampling line, an ionization chamber, which constitutes a secondary standard, is used to verify the values provided by the test unit and their stability. Its lower sensitivity limit is a few Bq·m^−3^ for ^222^Rn.
